# Perceptions of Canadian Radiation Oncologists, Medical Physicists, and Radiation Trainees about the Feasibility and Need of Boron Neutron Capture Therapy (BNCT) in Canada: A National Survey

**DOI:** 10.3390/cancers15143626

**Published:** 2023-07-14

**Authors:** Al-Retage Al-Bader, John Agapito, Ming Pan

**Affiliations:** 1 Schulich School of Medicine and Dentistry, University of Western Ontario, Windsor, ON N9B 2Y9, Canada; 2 Windsor Regional Hospital, 1995 Lens Ave, Windsor, ON N8W 1L9, Canada; 3 Department of Physics, University of Windsor, Windsor, ON N9B 3P4, Canada

**Keywords:** boron neutron capture therapy, radiation oncology, medical physics, Canada, national survey

## Abstract

**Simple Summary:**

Technological advancements in accelerator-based neutron sources have allowed for a more accessible and feasible study of Boron Neutron Capture Therapy (BNCT). Accelerator-based BNCT centers are being developed world-wide, and recent research is showing the potential in treating cancers. There are ongoing efforts to initiate Canadian contributions to BNCT research and the development of a Canadian center. We surveyed radiation oncologists and medical physicists in Canada to study their perception, understanding, and support for BNCT. The results of this survey guide Canadian contributions by identifying knowledge gaps and collaborative opportunities that support the success of this innovative cancer treatment in Canada.

**Abstract:**

Background: Boron Neutron Capture Therapy (BNCT) is an emerging radiotherapy. There are ongoing efforts to develop a Canadian accelerator-based BNCT center. However, it remains unclear how Canadian radiation oncologists (RO), medical physicists (MP), and their trainees perceive BNCT and its impact on radiation oncology as a discipline. Methods: A survey was created to explore the knowledge of BNCT, its clinical role, and the support for Canadian research. It was distributed through the Canadian Association of Radiation Oncology (CARO) and the Canadian Organization of Medical Physicists (COMP). Results: We received 118 valid responses from all 10 provinces, from 70 RO (59.3%) and 48 MP (40.7%), including 9 residents. Most knew of BNCT and its indications (60.2%). Although many were unaware of the reasons behind early failures (44.1%), common reasons were a lack of clinical trials and an inaccessibility of neutron sources (42.4%) as well as reactor unsuitability (34.7%). Additionally, 90.6% showed definite (66.9%) or possible (23.7%) support for Canadian BNCT research, while 89% indicated a definite (56.8%) or possible (32.2%) willingness for BNCT referrals. Conclusions: Most ROs and MPs supported Canadian BNCT research and would refer patients. However, limited awareness and a lack of experiences remain a challenge. Educational sessions are needed to realize this innovative cancer treatment in Canada.

## 1. Introduction

### 1.1. BNCT’s Emerging Role in Radiotherapy

As oncological diseases continue to be a leading cause of morbidity and mortality in Canada, efforts continue to discover, validate, and refine existing and emerging treatment options [1]. Boron Neutron Capture Therapy (BNCT) is an emerging radiotherapy technique, consisting of a neutron capture reaction from boron.

Boron carrier agents, such as sodium borocaptate (BSH) and boronophenylalanine (BPA), are administered to patients to create a higher concentration of boron in tumor cells compared to normal tissue [2,3,4]. An exposure to neutron beams causes localized neutron capture reactions which release high linear energy transfer (LET) alpha particles that generate many ionizations over a cellular distance of <10 µm, causing tumor cell death [5].

Compared to standard radiotherapy, BNCT’s advantage is its selectivity toward cancer cells, minimizing the risk of side effects to normal and radiosensitive tissue. Its efficacy depends on an appropriate boron concentration in tumor cells and neutron beam energy characteristics [6]. Early BNCT studies have relied on neutron sources from nuclear reactors, which was a significant limiting factor in BNCT’s research uptake and clinical consideration. However, recent technological advancements in accelerator-based neutron sources increased global momentum and the interest in BNCT.

### 1.2. BNCT History

Its theory was proposed in 1936 and tested in 1951 in the Brookhaven National Laboratory (BNL) and Massachusetts Institute of Technology (MIT) labs with limited success [7,8]. In the coming decades, efforts continued to refine BNCT through appropriate boron compounds [3,4] and neutron beam characteristics [9]. This allowed for clinical trials and prospective testing on various cancers, such as glioblastoma (GBM), head and neck cancers, and melanoma [5]. Japan, Finland, and the USA have been active in this regard, with studies demonstrating BNCT’s promising potential to be an efficacious and safe therapy. For example, over 300 BNCT treatments for GBM and head and neck cancers have been carried out between 1999 and 2012 in the FiR 1 research reactor in Otaniemi (Espoo, Finland) [9]. Additionally, a phase 1/2 trial of locally recurrent and inoperable head and neck cancers in 30 patients was conducted, resulting in an overall response rate of 76% and a 2-year survival of 30% [10]. Furthermore, Miyatake et al. applied BNCT to 167 patients with glioblastoma from 2002 to 2014, reporting a median overall survival of 10.8 months for recurrent tumors and 15.6 months for newly diagnosed glioblastomas [11]. While such work sustained interest in BNCT, its reliance on almost inaccessible neutron sources such as nuclear reactors limited large-scale clinical trials.

### 1.3. Accessibility of Neutron Sources

The previous reliance on nuclear reactors posed significant barriers to research and clinical applications. Challenges arose due to high costs, unsuitable infrastructure for hospitals and patient use, uranium fuel’s environmental impacts, and broad neutron beam characteristics [6,12]. Canada also faces a limited availability of neutron sources, particularly after the closure of the National Research Universal (NRU) Reactor in Chalk River in 2018 [13].

The need for accessible neutron sources propelled advances in a compact accelerator-based neutron source (CANS) which eventually succeeded [11,14,15,16]. In 2016, the first accelerator-based BNCT (AB-BNCT) facility was built in Japan’s Kyoto University by Sumitomo Heavy Industries [6,17,18].

The development of CANS renewed interest in BNCT and facilitated the development of AB-BNCT centers in various countries such as Japan, the UK, China, Finland, and Russia [19,20,21]. Importantly, it also helped demonstrate the clinical value of AB-BNCT, as Japan became the first country to gain approval and insurance coverage for the use of BNCT in recurrent head and neck carcinoma, indicating a significant milestone in BNCT history [6,22]. Additionally, the International Atomic Energy Agency (IAEA) has been engaged in updating the now outdated 20-year-old technical guideline document [23]. Taken together, there has been concerted global interest and effort to further understand BNCT and its clinical potential, which requires a large-scale multidisciplinary collaboration of international stakeholders to ensure its success.

### 1.4. Objectives

Despite global interest in BNCT research, Canada has yet to play a major role in this field. To support Canadian contributions, a Canada Foundation for Innovation (CFI) 2023 Innovation Fund is underway (Project number: 42891) to develop a low-cost prototype Canadian compact accelerator-based neutron source (PC-CANS) [13,24,25]. This technology would be intended to apply neutron-based research techniques including BNCT [13].

However, there has been limited exploration of the recent perspectives and of the knowledge status of key stakeholders that could potentially be involved in future BNCT research and therapy. Such perspectives are important to account for in the context of recent technological advancements and innovation.

Our objective is to explore the perceptions and understanding of BNCT amongst Canadian radiation oncologists (RO), medical physicists (MP), and their resident trainees. Specifically, a survey was distributed to explore the following: (1) understanding of BNCT’s history and recent developments, (2) support towards Canadian BNCT research, and (3) recognition of clinical applications of BNCT.

We hypothesized varying perceptions ranging from support towards BNCT, considering its recent developments and global uptake, in addition to some hesitations due to limited large-scale clinical data. The results of this survey guide Canadian contributions by identifying knowledge gaps and collaborative opportunities that support the success of this innovative cancer treatment in Canada.

## 2. Methods

After an in-depth scientific review of the literature, a survey was created using Google Survey. The survey was distributed through two national organizations, i.e., the Canadian Association of Radiation Oncology (CARO) and the Canadian Organization of Medical Physicists (COMP). Informed consent was obtained prior to participation in the study. This study was approved by the Windsor Regional Hospital Research Ethics Board (REB #22-429) as well as the Board of Directors of both CARO and COMP.

The survey was open to collect responses between 24 January 2022 and 23 May 2022. It was voluntary, without compensation, and participants remain anonymous. There was a total of 17 items, divided into 3 domains: (1) eligibility, (2) demographics, and (3) specific knowledge of BNCT and utilization. For some questions, respondents had the ability to offer their own responses, in addition to selecting existing options. Respondents also had the opportunity to offer final comments or opinions in the last item of the survey. There were no excluded responses. The results were analyzed using descriptive statistics and IBM SPSS Statistics, Version 28.0.1.1(14).

## 3. Results

### 3.1. Eligibility and Response Rate

Eligibility is limited to ROs with an independent/academic license, board-certified MPs, or residents in a formal residency-training program. A total of 118 respondents were collected: 53.4% (N = 63) were radiation oncologists, 39% (N = 46) were medical physicists, while 5.9% (N = 7) were residents in radiation oncology and 1.7% (N = 2) were residents in medical physics. There were no missing answers throughout the survey.

As of May 2022, the survey was distributed by CARO to 305 ROs, 56 MPs, and 231 residents (including international clinical fellows). It is known that many of CARO’s MP members also have a COMP membership. At the same time, COMP has distributed the survey to 563 MPs and residents, but we do not know how many are foreign members or from other industries and not working in radiation oncology in Canada. Due to the limitation of defining domestic and international residents, it is impossible to know the real number of Canadian residents. However, based on the current Canadian postgraduate training positions, we estimated a maximum number of 105 Canadian residents who received the survey (i.e., there are only up to 21 such matching position per year in the 5-year RO training program nationwide). We are also unaware of the numbers of COMP’s Affiliate Members in other organizations and industry who are unlikely to be interested in our BNCT survey. As a result, we estimated the number of MP working or training in radiation oncology to be 50% of that of RO (i.e., about 150 MPs and 20 residents, given a 2-year residency training for a Canadian MP). Based on these assumptions, the response rates were estimated to be 21% for RO, 31% for MP, 7% for RO residents, and 10% for MP residents.

### 3.2. Demographics

Most respondents were between 35 and 45 years old (40.7%; N = 48) and male (72%; N = 85). Respondents’ practice region included all 10 Canadian provinces, with a majority from Ontario 45.8% (N = 54) followed by Quebec 18.6% (N = 22). Furthermore, most respondents reported being in practice for 10–20 years (30.5%; N = 36) followed by staff in practice for over 20 years (24.6%; N = 29). A full display of respondents’ demographics is displayed in Table 1.

### 3.3. Current Knowledge about BNCT

**Figure 1:**
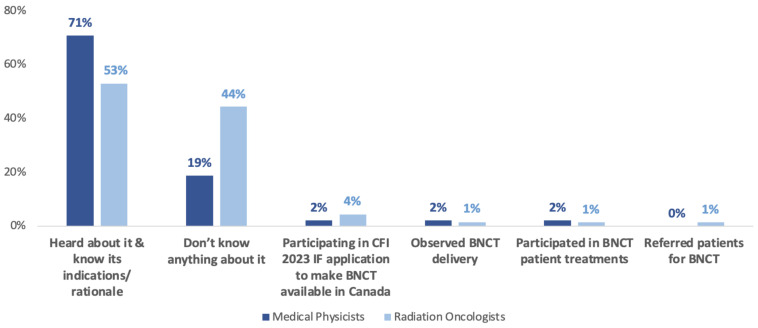
Current knowledge of BNCT in Medical Physicists (MP) and Radiation Oncologists (RO). Answer options are displayed in decreasing popularity.

Regarding knowledge about BNCT, the prevailing answer was that participants had previously heard about it and know about its indications and rationale (60.2%; N = 71; Table 2). Within subgroups, more MP knew about BNCT’s indications (70.8%; N = 34) compared to the RO group (52.9%; N = 37; Figure 1).

The second most common answer was that participants did not know about BNCT (33.9%; N = 40). Specifically, 44.3% of the RO group reported not knowing about BNCT (N = 31) compared to 18.8% of MPs (N = 9).

Only 4 respondents (3.4%) reported being involved in the CFI 2023 IF application to make BNCT available in Canada. A minority reported having encounters with BNCT in the form of participating in treatments (1.7%; N = 2) or referring patients to BNCT (0.8%; N = 1).

The additional responses that were offered mostly involved MPs indicating prior experiences in neutron physics technology development (1.7%; N = 2) and research activities (0.8%; N = 1). Additionally, 2 RO physicians reported hearing about it once or recalling it from training (Table 2).

### 3.4. Perceptions on the Failure of Early BNCT Studies

When probing for perceptions on reasons behind the lack of success of early BNCT research between the 1950s and 2000 in nuclear reactors, participants most frequently selected ‘I don’t know’ as a response (44.1%; N = 52). The second-most selected option referred to a lack of large clinical trials related to the limited availability of neutron sources or BNCT centers (42.4%; N = 50).

**Figure 2:**
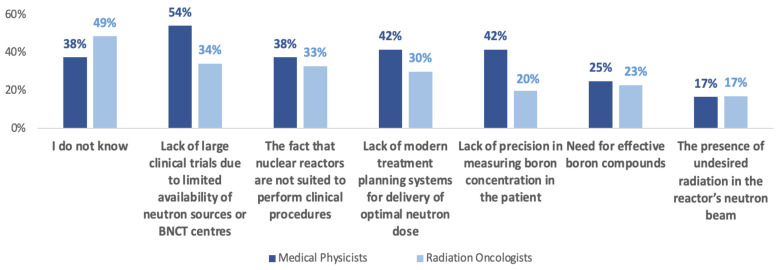
Perceptions on the lack of success of early BNCT studies between 1950 and 2000, depending on occupation. Answer options are displayed in decreasing popularity.

When comparing differences between RO and MP perceptions, the biggest difference was found in the number of respondents selecting ‘lack of precision in measuring boron concentration in the patient’ as an option. Specifically, 41.7% of physicists (N = 20) selected this option compared to 20% of oncologists (N = 14; Figure 2).

Participants also had the ability to offer responses, most commonly pointing towards a lack of infrastructure (2.5%; N = 3) such as “no availability of reactors” and “no linac-based neutron sources” (Table 3).

### 3.5. Radiation Oncologists’ Treatment Recommendations

For the four questions in this section, only radiation oncologists’ answers were analyzed to accurately reflect the reality of clinical decision making (N = 70). In addition to the listed choices, respondents had the ability to offer additional written responses, detailed in Table 4.

The cases included recurrent and unresectable tumors following maximal dose chemoradiation. The most common treatment recommendations selected were as follows: palliative chemo such as temozolomide (68.6%; N = 48) for GBM, supportive care only (38.6%; N = 27) for malignant meningioma, palliative chemotherapy (67.1%; N = 47) for head and neck cancer, and fourth line targeting therapy or immunotherapy (61.4%; N = 43) for localized unresectable malignant melanoma.

Out of seven listed options, BNCT was the sixth most rated option for glioblastoma (17.1%; N = 12), in addition to malignant melanoma and meningioma (15.7%; N = 11). For head and neck cancers, BNCT was the least popular option compared to the other listed options (18.6%; N = 13).

### 3.6. Awareness of Current BNCT Development

**Figure 3:**
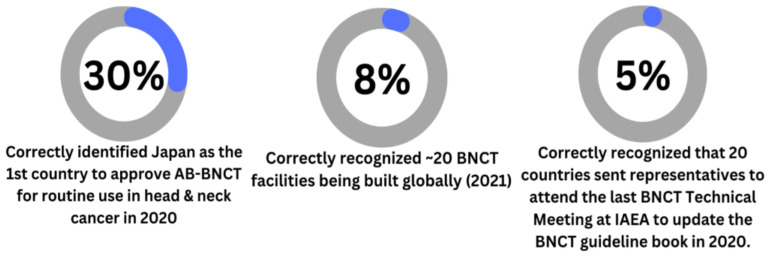
Medical Physicists and Radiation Oncologists’ Awareness of Current BNCT Development.

This section of the survey included three questions regarding recent significant milestones in BNCT history to further understand the knowledge status of RO and MP in regards to recent developments (Figure 3). The survey showed that 29.7% (N = 35) of the total respondents correctly identified Japan as the first country to approve AB-BNCT for routine use in head and neck cancer in 2020 [26]. Additionally, 7.7% (N = 9) correctly recognized that there are about 20 BNCT facilities being built globally as of 2021 [27]. Lastly, 5.1% (N = 6) of respondents correctly recognized that 20 countries sent representatives to attend the last BNCT Technical Meeting at IAEA to update the BNCT guideline book in 2020 [26]. In contrast, 66.1–89.8% (N = 78–106) of respondents indicated that they did not know the answers to these questions.

### 3.7. Opinions on Joining BNCT Research and in the Clinical Context

**Figure 4:**
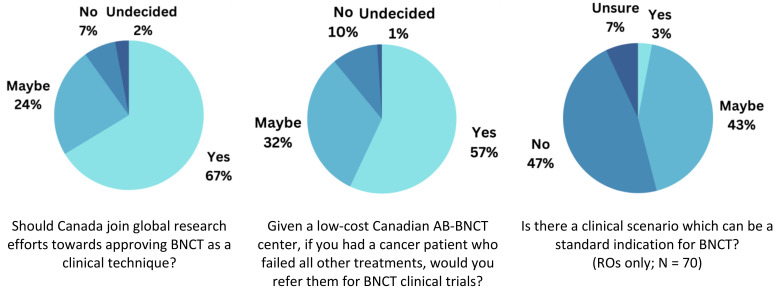
Overall opinions on BNCT research and BNCT in the clinical context.

Respondents were asked for their interest in supporting Canadian research efforts, their perspectives on referrals, and their recognition for BNCT’s clinical role, demonstrated in Figure 4.

When asked whether Canada should join interdisciplinary global research efforts to move BNCT towards a clinical technique, 66.9% (N = 79) of the total respondents agreed. Additionally, 23.7% (N = 28) selected ‘maybe’, meaning they would possibly support BNCT research efforts. A minority of respondents (6.8%; N = 8) disagreed with joining research. For this question, overall responses between MPs and ROs were comparable, as 68.8% (N = 33) of physicists and 65.7% (N = 46) of oncologists similarly agreed to join research efforts.

As for the willingness to refer cancer patients who failed all other treatments, should there be a low-cost AB-BNCT facility in Canada, a total of 56.8% of respondents (N = 67) agreed, while 32.2% (N = 38) indicated that they may refer. Alternatively, 10.2% (N = 12) would not refer. Comparing between groups, slightly more radiation oncologists would refer to BNCT (61.4%; N = 43) compared to physicists (50%; N = 24), while more physicists (37.5%; N = 18) selected ‘maybe’ compared to oncologists (28.6%; N = 20)

The last question asks for possible clinical scenarios that could be considered a standard indication for BNCT. Data analysis focused only on the RO respondents (N = 70) to best reflect the reality of clinical practice, as most MP provided invalid answers suggesting that they would not typically make such decisions. However, only one physicist agreed (GBM). Two oncologists (2.9%) suggested “advanced sarcoma after debulking surgery, possibly hypernephroma with 10BPA” and “Recurrent HNC, GBM, malignant meningioma, melanoma that are unresectable after maximal dose EBRT”. In contrast, 42.9% (N = 30) of RO physicians suggested the possibility of a standard indication but did not offer examples, while 47.1% (N = 33) disagreed.

### 3.8. Additional Comments

Participants had the ability to anonymously offer additional comments to express overall opinions on BNCT. Comments included hesitations and enthusiasm towards BNCT, interest in research, lack of awareness of BNCT, and practical considerations of BNCT research.

#### 3.8.1. Hesitations towards BNCT Value

Some respondents expressed reservations: the premise of using BNCT after radiotherapy “is a bit flawed [and] would have to be better supported”. Some expressed hesitations due to the slow progression: “[I’m] wary about investing time and effort in developing a technique that hasn’t received substantial uptake despite decades of existence” and “we’ve pushed the dose confinement paradigm for the last 70 years without seeing a huge improvement in cure although toxicity has been greatly reduced… I think the future lies with targeted immunotherapy…. I am prepared to keep an open mind, but I would need convincing to put research dollars into this enterprise”. More specifically, one voiced concern about the limited knowledge on “an optimal Boron compound [and] the optimal administration [and] resulting tumor/tissue uptake”.

#### 3.8.2. Enthusiasm towards the Potential of Canadian BNCT Research

Alternatively, affirming the Canadian potential to join and succeed in BNCT research, respondents offered the following: “I think we should move forward with BNCT; often the introduction of a new technique serves as a catalyst for new discoveries” and “BNCT shows interesting applications in advanced forms of cancer that have limited curative options. I’m very happy to see a Canadian initiative to bring this modality!” Another highlighted Canada’s capabilities, saying, “we have knowledge, the resource, and clinical science background. We should move forward.”

#### 3.8.3. Considerations of BNCT Research

Regarding the nature of BNCT research, some commented on Canada’s “expertise to handle a project of this nature” and “capabilities of performing comprehensive clinical trials with the appropriate number of patients.” A comment noted BNCT as a “purely investigational therapy,” but it is still worth “participating in developing and evaluating promising investigational therapies.” Another commented, having “missed the opportunity to develop Proton Therapy, [Canada needs to] catch up the other particle radiation therapy research to join the international pioneers to advance our specialty and improve cancer patients’ outcome.”

Furthermore, there were suggestions of the need to consider “travel, accommodation and incidental costs” as a potential barrier for referral, especially for patients in rural areas. Moreover, given the multidisciplinary nature of such research, the importance of “collaboration between high-level radiation oncologists, medical physicists, radiobiologists, and medical/radiation chemists” was highlighted, requiring “institutions [capable of] large scale clinical trials support,” such as those “located in regions of large patient populations.”

#### 3.8.4. Limited Awareness of BNCT

Lastly, there were an indication and an interest for continued education of BNCT in academic circles: “I am not familiar with it, but I can see the benefit if properly implemented” and “I know next to nothing about BNCT. It would be great to see some talks on the subject at the next COMP meeting.”

## 4. Discussion

This survey explores the perspectives and the knowledge status of Canadian medical physicists and radiation oncologists regarding BNCT to guide future Canadian contributions.

Our results show that the majority are aware of BNCT and its indications (60.2%) and attribute the lack of success of early BNCT studies to limited trials (42.4%) and neutron source inaccessibility (34.7%). However, a large proportion do not know about BNCT (33.9%), which aligns with 44.1% not knowing reasons behind its failure and only 5.1–29.7% correctly identifying recent developments and global practices. Overall, 90.6% showed definite (66.9%) or possible (23.7%) support for Canadian BNCT research, while 89% indicated a definite (56.8%) or possible (32.2%) willingness for BNCT referrals.

In the context of ongoing efforts for Canadian contributions to BNCT, the survey results demonstrate support for a PC-CANS to support AB-BNCT research and clinical application [13,24]. This would align with Canada’s rich history as a leader in materials science and neutron physics, an example of which includes its pivotal role in introducing Cobalt-60 for cancer radiotherapy at Victoria Hospital (London, Ontario) in 1951 [13,28].

### 4.1. Willingness for BNCT Referrals

A majority (89%) of respondents showed definite (56.8%) or possible (32.2%) willingness to refer patients to Canadian BNCT facilities despite a small minority having direct interactions with BNCT referrals (0.8%; N = 1). Additionally, two comments noted barriers and access to centers as important considerations in the context of referrals, especially for patients in rural areas. This concern is consistent with the previous literature indicating barriers to access to radiotherapies in Canada as being patient age (34.7%), distance to centers (30.7%), wait times (29.3%), and provider factors such as a lack of understanding about the use of radiotherapy (21.6%) [29].

The overall willingness for BNCT referrals aligns with the recommendations of BNCT by 15.7–18.6% of ROs in the provided clinical scenarios (glioblastoma, head and neck cancer, meningioma, and melanoma). This is supported by clinical trials showing BNCT’s clinical potential in treating cancers; a further review of clinical applications can be found in Malouff et al. (2021) and Moss (2014) [5,12]. Although the literature suggests that BNCT is a safe and efficacious treatment modality, large-scale trials are needed for more conclusive data, accounting for the existing literature’s varying inclusion criteria, cancer subtypes, boron compounds and their administration, and BNCT delivery techniques and neutron doses. There is also a need for Phase 2 and Phase 3 trials to compare BNCT against standard therapies to further validate its safety and efficacy [5,6,30].

For example, when asked about barriers limiting BNCT’s success, survey results indicated that limited clinical trials and infrastructure unsuitability were weighted more heavily than planning systems and concerns with boron agents and dosimetry. This is consistent with assertions in the literature highlighting the essential role of clinical trials and neutron source accessibility to sustain interest and progress in BNCT and their lack thereof as the biggest weakness limiting BNCT [12,19].

### 4.2. Unanswered Questions in BNCT

Despite significant progress in the field of BNCT, research efforts continue to optimize BNCT’s delivery and outcome. Continuing areas of research focus on treatment planning and optimized delivery of existing or new boron carrier agents. For example, efforts continue to further understand second-generation BPA’s and BSH’s biodistribution profiles [23,31]. Moreover, there is work on developing third-generation boron compounds with more active uptake mechanisms such as boronated biologics including boronated DNA intercalators, peptides, etc. [6]. Furthermore, recent research exploring cerebrospinal administration of BPA for brain tumors has been underway [32]. Additionally, there has been a reported survival advantage in GBM for longer infusion times (6 h) of BPA (6 h vs. 2 h) [33]. Collectively, this suggests the possibility for optimized use of existing compounds. However, such endeavors have been limited by a lack of neutron sources and accelerators for validation and testing, which has led to minimal uptake and incentive for pharmaceutical industry support [12]. The implementation of AB-BNCT centers and concerted global trials/research would support progress in answering these questions.

### 4.3. Limited Knowledge on BNCT

A recurring theme throughout the results points to limited knowledge on BNCT’s current status, reasons for a lack of success, and recent developments and applications. For example, 33.9% indicated they do not know about BNCT, which was more pronounced in RO (44.3%) compared to MP (18.8%). Additionally, 44.1% indicated that they did not know the reasons behind the lack of success of early BNCT studies, and only 5–29.7% were able to correctly identify recent developments and BNCT practices. This is also supported by the minority who have had direct interactions with BNCT through treatment observations (1.7%) and participation in BNCT treatments (1.7%) or referrals (0.8%).

The literature has also suggested that the lack of awareness of neutron therapies from the public and providers are related to negative perceptions surrounding nuclear reactors and radiation [15]. Specifically, a series of nuclear disasters such as the Fukushima nuclear disaster in 2011 [15] and radioactive leakages prior to the Chalk River shutdown may account for hesitations and a lack of awareness. This highlights the importance of healthcare providers’ correct understanding of this technique and its developments for its progression in research and clinical application. This is especially vital given the highly multidisciplinary collaboration required. Hopefully, the new IAEA’s BNCT guideline will help solve the knowledge gap [34].

Collectively, our survey results give quantitative confirmation on the proposed limited awareness of BNCT, particularly amongst Canadian radiation oncologists and medical physicists. This would have to be considered in the context of Canadian research initiatives and building of an AB-BNCT facility and point towards the value of educational activities [13,24].

### 4.4. Limitations

There are some limitations in this survey. The results may be susceptible to bias recruitment, as those most interested in BNCT may be more inclined to respond to the survey and represent support. Additionally, despite overall representation from all 10 Canadian provinces, most respondents were from Ontario (45.7%) and Quebec (18.6%). Although Ontario and Quebec are the most highly populated provinces, which have 61% of the Canadian population, more feedback from other provinces would further affirm if such notions are common and widely held throughout Canada.

## 5. Conclusions

With recent technological advancements in accelerator-based neutron sources allowing for accessible AB-BNCT centers, there is renewed global interest in BNCT research. The results of this survey demonstrate that most Canadian ROs and MPs support Canadian BNCT research efforts and would refer patients to a Canadian BNCT center. The implications of such results encourage Canadian participation in BNCT research and the construction of the first Canadian AB-BNCT facility. However, the limited knowledge about BNCT poses a challenge and highlights the value of educational sessions about BNCT to successfully realize this goal.

## Figures and Tables

**Table 1:** Respondent demographics, by occupation.

	Medical Physicists (40.7%; N = 48)	Radiation Oncologists (59.3%; N = 70)	Total % (N = 118)
**Age**			
≤35 Years	7	12	16.1%
>35 Years, and ≤45 years	22	26	40.7%
>45 Years, and ≤55 years	9	13	18.6%
>55 Years, and ≤65 years	6	10	13.6%
>65 Years	3	5	6.8%
Prefer not to say	1	4	4.2%
**Gender**			
Male	35	50	72%
Female	10	16	22%
Prefer not to say	3	4	5.9%
**Region of Practice**			
Ontario	20	34	45.8%
Quebec	10	12	18.6%
British Columbia	2	5	5.9%
Saskatchewan	4	3	5.9%
Nova Scotia	1	4	4.2%
Alberta	2	4	5.1%
Manitoba	1	1	1.7%
New Brunswick		1	0.8%
Newfoundland and Labrador		1	0.8%
Prince Edward Island	2	1	2.5%
Canada	1		0.8%
Outside Canada	5	4	7.6%
**Years in Practice**			
>10 Years, and ≤20 years	16	20	30.5%
>20 Years	12	17	24.6%
>5 Years, and ≤10 years	11	11	18.6%
0–5 Years	7	15	18.6%
I am a resident	2	7	7.6%

**Table 2:** Current knowledge of BNCT (bolded options are those listed on the survey and others are free-text answers).

Current Knowledge Status of BNCT	Number of Respondents	% of Total Respondents (N = 118)
**I have heard about it and know its indications or rationale**	71	60.2%
**Don’t know anything about it**	40	33.9%
**I am participating in the CFI 2023 IF application to make BNCT available in Canada**	4	3.4%
**I have observed the delivery of BNCT**	2	1.7%
**I have participated in BNCT patient treatments**	2	1.7%
I have experience in neutron physics and BNCT related technology devlopment	2	1.7%
I have read about it	2	1.7%
**I have referred patients for BNCT**	1	0.8%
Heard about it once	1	0.8%
I have engaged in or published research on BNCT	1	0.8%
I recall learning about it during training	1	0.8%

**Table 3:** Overall perceptions on the lack of success of early BNCT studies between 1950 and 2000. (Bolded options are those listed on the survey, and others are free-text answers).

Reasons for Failure of Success of Early BNCT Studies between 1950–2000	Number of Respondents	% of Total Respondents (N = 118)
**I do not know**	52	44.1%
**Lack of large clinical trials due to limited availability of neutron sources or BNCT centres**	50	42.4%
**The fact that nuclear reactors are not suited to perform clinical procedures**	41	34.7%
**Lack of modern treatment planning systems for delivery of optimal neutron dose**	41	34.7%
**Lack of precision in measuring boron concentration in the patient**	34	28.8%
**Need for effective boron compounds**	28	23.7%
**The presence of undesired radiation in the reactor’s neutron beam**	20	16.9%
Lack of infrastructure	3	2.5%
Need for precise dosimetry information	2	1.7%
Budget vs benefits/risks	1	0.8%
Insufficient efficacy data to merit overcoming logistical barriers associated with theranostic interventions	1	0.8%
Lack of motivation from current centers to develop proper evidence	1	0.80%
Relative ease and availability of IMRT and not cost effective by comparison	1	0.80%
Probably a combination of multiple above	1	0.80%

**Table 4:** Radiation oncologists’ (N = 70) treatment recommendations for unresectable cancers that recurred/progressed after a maximal-dose chemoradiation (assuming there is an institution close-by providing BNCT or doing clinical trials; bolded options are those listed on the survey).

Unresectable Cancers	Number of Respondents	% of Respondents (N = 70)
**Glioblastoma** (60 years old, maximal dose chemoradiation 6000 cGy or higher)		
**1. Palliative chemo such as temozolomide**	48	68.6%
**2. Supportive care only**	41	58.6%
**3. Bevacizumab or other systemic agents**	33	47.1%
**4. SRS**	16	22.9%
**5. Palliative low dose external beam radiation**	13	18.6%
**6. BNCT**	12	17.1%
**7. I do not know**	10	14.3%
8. Clinical trials	5	7.1%
9. Re-irradiation	2	2.9%
10. Tumor Treating Fields	2	2.9%
11. Refer to CNS colleague	1	1.4%
12. BCNU	1	1.4%
**Malignant Meningioma** (50 years old, maximal dose chemoradiation 6000 cGy or higher)		
***1. Supportive care only***	27	38.6%
***2. Immunotherapy or other systemic agents***	25	35.7%
***3. I do not know***	22	31.4%
***4. SRS***	19	27.1%
***5. Palliative chemo***	17	24.3%
***6. BNCT***	11	15.7%
***7. Palliative low dose external beam radiation***	10	14.3%
8. Clinical trials	3	4.3%
9. Referral for outside opinion	2	2.9%
10. Surgery	1	1.4%
**Head and Neck** (65-years old, maximal dose chemoradiation 7000 cGy or higher)		
***1. Palliative chemo***	47	67.1%
***2. Immunotherapy or other systemic agents***	47	67.1%
***3. Supportive care only***	35	50%
***4. Palliative low dose external beam radiation***	33	47.1%
***5. I do not know***	22	31.4%
***6. SRS or SBRT***	18	25.7%
***7. BNCT***	13	18.6%
8. Re-irradiation with IMRT	1	1.4%
9. Repeat radical RT may be feasible.	1	1.4%
10. Refer to H&N colleague	1	1.4%
11. Depends on region of recurrence and extent, local vs distal and performance status	1	1.4%
**Melanoma** (30 years old, recurred after multiple surgeries, high dose adjuvant radiation therapy, and third line systemic treatment had to stop due to severe toxicity)		
***1. Fourth line targeting therapy or immunotherapy***	*43*	*61.4*%
***2. Supportive care only***	*36*	*51.4*%
***3. Palliative low dose external beam radiation***	*23*	*32.9*%
***4. SRS or SBRT***	*20*	*28.6*%
***5. Palliative chemo***	*19*	*27.1*%
***6. BNCT***	*11*	*15.7*%
***7. I do not know***	*7*	*10*%
8. Clinical trials	7	10%
9. Embolization	1	1.4%

## Data Availability

Not applicable.
